# Association between Food Sources of Free Sugars and Weight Status among Children and Adolescents in Japan: The 2016 National Health and Nutrition Survey, Japan

**DOI:** 10.3390/nu14173659

**Published:** 2022-09-04

**Authors:** Aya Fujiwara, Emiko Okada, Mai Matsumoto, Ryoko Tajima, Xiaoyi Yuan, Hidemi Takimoto

**Affiliations:** 1 Department of Epidemiology and Prevention, Center for Clinical Sciences, National Center for Global Health and Medicine, Tokyo 162-8655, Japan; 2 Department of Nutritional Epidemiology and Shokuiku, National Institutes of Biomedical Innovation, Health and Nutrition, Tokyo 162-8636, Japan; 3 Department of Social and Preventive Epidemiology, School of Public Health, University of Tokyo, Tokyo 113-0033, Japan

**Keywords:** free sugars, food sources, solids and liquids, weight status, BMI, overweight and obesity, national survey, Japanese, children and adolescents

## Abstract

This cross-sectional study aimed to investigate the relationship between food sources of free sugars and weight status among children and adolescents aged 2–19 years in Japan (1438 males and 1340 females) from the 2016 National Health and Nutrition Survey, Japan. We estimated the percentage of energy intake (% E) from free sugars from total food sources (FS_total_), solid foods (FS_solids_), and beverages (FS_liquids_), using one-day weighed dietary record data. Weight status was determined based on body mass index (BMI) z-scores and the prevalence of overweight and obesity. The mean energy intakes of FS_total_, FS_solids_, and FS_liquids_ were 5.8%, 4.1%, and 1.8% in males and 6.2%, 4.6%, and 1.6% in females, respectively. After controlling for potential confounding factors, including energy intake, there was no association of free sugars intake from all food sources with BMI z-scores or with the prevalence of overweight and obesity, except for a marginal association between higher FS_solids_ and lower estimated BMI z-scores in females (*p* = 0.05). The present findings indicate that among children and adolescents in Japan, who have a relatively low mean intake of free sugars, consuming free sugars from any food source is unlikely to have an adverse effect on weight status.

## 1. Introduction

The rising obesity pandemic among children and adolescents has attracted attention worldwide. In 2015, 5.0% (107.7 million) of children and adolescents were obese, and the prevalence was greater in high-income countries [1]. Although Japan has a lower prevalence of obesity (≤3%) among children and adolescents than in other high-income countries, the prevalence has increased between 1980 and 2015 [1]. Weight status during the early stages of life has prolonged effects on weight status [2], morbidity, and mortality [3] later in life. Therefore, it is necessary to identify modifiable risk factors for obesity, such as diet, in the early stages of life to improve long-term health.

The World Health Organization (WHO) and many countries have recommended reducing the intake of sugars added during manufacturing and cooking (i.e., added sugars or free sugars), not naturally occurring sugars, to prevent unhealthy weight gain in children and adolescents owing to excess energy intake from sugar [4,5]. The existing evidence on the relationship between dietary sugars and weight gain is mainly derived from studies investigating the effects of sugars from soft drinks [6,7]. However, sugars from different food sources, such as solid foods and beverages, may have different effects on weight status [8,9]. Few studies have investigated the influence of different food sources on weight status, and the results of these studies have been inconsistent. In Australia, a cross-sectional study among children and adolescents aged 2–16 years found no associations between body mass index (BMI) z-scores and free sugars intake (the sum of added sugars and sugars from fruit juices and fruit juice concentrates [10]) from total food sources, solid food, and beverages [11]. In contrast, in another cross-sectional study among children and adolescents aged 2–19 years in America, it was reported that consuming added sugars from solid foods was inversely associated with weight status while consuming those from beverages was positively associated [12]. In Canada, similar associations between consuming added sugar from different food sources and BMI were reported following a cohort study among children aged 8–10 years at baseline [13]. However, consuming added sugars from neither solid foods nor beverages was associated with BMI changes during the 2-year follow-up period in the same population [14].

A possible mechanism explaining the different effects of various food sources of free sugars on weight status is poorer satiety and incomplete compensation, as well as a rapid increase in blood glucose from beverages compared with solid foods, resulting in an increased energy intake and subsequent weight gain [8,9]. In contrast, as widely consumed beverages [15], soft drinks may act as a marker of an unhealthy lifestyle (for example, unhealthy dietary habits [16,17]) that can increase an individual’s susceptibility to weight gain. To our knowledge, information on the relationship between dietary sugars and weight status among children and adolescents in Asian countries, including Japan, is scarce. Although previous cross-sectional studies conducted in Japan have reported no association between free sugars from total food sources and BMI among children [18] and adolescents [19], the influence of different food sources have not been considered. Considering the relatively lower intake of free sugars and soft drinks among children and adolescents in Japan compared with their counterparts in Western countries [20], this association would likely differ between Japan and Western countries.

This cross-sectional study aimed to investigate the relationship between consuming free sugars from different food sources and weight status among children and adolescents aged 2–19 years in Japan, using one-day dietary data from the 2016 National Health and Nutrition Survey (NHNS), Japan.

## 2. Materials and Methods

### 2.1. Data Source and Analytic Sample

This cross-sectional analysis was based on data from the 2016 NHNS and was conducted with permission from the Ministry of Health, Labor and Welfare (MHLW). Details of the survey have been described elsewhere [21,22,23]. Briefly, the NHNS is a national household survey that annually assesses the dietary intake, lifestyle, and health conditions of household members. Data from the 2016 survey was used for the present analysis owing to it having a much larger sample size than that of more recent studies. Based on the 2010 national census data, a total of 462 census units were sampled as survey areas. Participants were all non-institutionalized, from Japan, aged ≥1 year (as of 1 November 2016), and living in the survey areas. Those excluded (the number was unknown) from the survey were households in which the heads were foreign citizens, individuals without a self-selected diet, and individuals on a special diet (mainly owing to disease). The survey was conducted between October and November 2016. Of the 2,4187 eligible households, 1,0745 (44.4%) participated in the final survey. The survey was conducted according to the guidelines in the Declaration of Helsinki, and verbal informed consent was obtained from all participants. Here, we used anonymized individual-level NHNS data provided by the MHLW for secondary use. Thus, this analysis was exempt from the requirement for ethical review and institutional review board approval [24].

Of the total participants in the 2016 NHNS (*n* = 3,0820), 4380 participants were aged 2–19 years. For this analysis, all participants who completed the dietary survey (*n* = 4085) were included. Pregnant female participant (*n* = 1) and those without body height or weight data (*n* = 1306) we excluded. The final sample used in this analysis included 1438 males and 1340 females.

### 2.2. Dietary Assessment

Dietary intake was assessed using a one-day weighed household dietary record on any day other than Sunday, national holidays, and days with special events [21,22,23]. Data on all members of each household were included in the analysis. Briefly, the main household food preparer (referred to as the record keeper) was asked to weigh and record all food and beverages consumed by household members on the recording day. If household members shared food items from a single dish, the record keeper was asked to record the approximate proportions of food consumed by each member and the number of leftovers. When the record keepers encountered any difficulty with weighing, such as eating out or having school-prepared lunch, they were asked to document as much information as possible, including the estimated portion sizes and details of leftovers. Trained fieldworkers then checked the records. If necessary, the trained fieldworkers collected additional information from the record keepers and revised the records. Thereafter, the trained fieldworkers estimated the weights of the foods and beverages consumed according to the portion sizes recorded using household measures. When participants ate a school-prepared lunch, the trained fieldworkers were asked to obtain the lunch menus and information on the names and weights of the ingredients used from each school. These were then used to estimate the weights of the food and beverages consumed. Thereafter, the trained fieldworkers assigned food codes to all the items based on the NHNS study manual. At the local center, the trained fieldworkers captured the dietary intake data using special software developed for the NHNS. An overall dietary dataset was then compiled by trained investigators at the central office.

Using the household food consumption record and based on the Standard Tables of Food Composition in Japan (STFCJ) [25,26], the food and energy intake of each participant was estimated. Food grouping was performed based on the similarity in nutrient profiles and culinary usage according to the STFCJ [25,26]. According to the WHO’s definition, free sugars are defined as sugars added by the manufacturer, cook, or consumer, as well as sugars naturally present in honey, syrups, fruit juices, and fruit juice, concentrates [10]. Free sugars intake [10] was estimated using a recently developed comprehensive food composition database [20] for commonly consumed food items in Japan that are included in the STFCJ [25,26]. The intake of free sugars from solid foods and beverages (FS_solids_ and FS_liquids_) was estimated post-hoc by categorizing food sources as follows: FS_solids_ was defined as the intake of free sugars from all foods, including semi-solid foods such as yogurt and soups. Free sugars added to tea and coffee at the table were also included as FS_solids_ because they could not be differentiated from free sugars added during cooking in the household dietary record. FS_liquids_ was defined as the intake of free sugars from beverages such as fruit and vegetable juices, alcoholic beverages, and soft drinks (i.e., soda, sports drinks, fruit drinks, milk beverages, and pre-sweetened coffee).

The utility of the household dietary record was previously assessed based on individual-level dietary intake among the population of Japan [27]. Briefly, the dietary intake of young women (aged approximately 20 years) was assessed by comparing their own one-day household dietary records with that of their mothers (mean age, 49 years) (*n* = 32). The mean differences between the intakes, as determined by the two dietary records, for energy, protein, fat, and carbohydrates were 6.2%, 5.7%, 6.7%, and 6.3%, with Pearson correlation coefficients of 0.90, 0.89, 0.91, and 0.90, respectively [27].

### 2.3. Assessment of Weight Status

Information on sex and age was collected using a self-administered questionnaire. Trained fieldworkers conducted anthropometric measurements for 54.4% of the participants using standardized procedures. The participants wore light clothes and were barefoot when their body height (to the nearest 0.1 cm) and weight (to the nearest 0.1 kg) were measured. For the remaining 45.6% of the participants, height and weight were either measured at home by other household members or self-reported. BMI was calculated as weight (kg) divided by height squared (m^2^). Weight status was estimated using both BMI z-scores and the prevalence of overweight and obese participants. Briefly, BMI z-scores were calculated based on age- and sex-specific equations for ages 2–18 years using the International Obesity Task Force (IOTF) criteria [28]. The prevalence of overweight and obesity was either defined according to the IOTF age- and sex-specific BMI cut-off values [28], which correspond to an adult BMI of ≥25 kg/m^2^ (for participants aged <18 years), or based on the BMI cut-off value of ≥25 kg/m^2^ according to the WHO recommendations (for participants aged 18–19 years) [29].

### 2.4. Statistical Analysis

All statistical analyses were performed by sex using SAS statistical software, version 9.4 (SAS Institute Inc., Cary, NC, USA). All reported *p*-values were two-tailed, and statistical significance was set at *p* < 0.05. Descriptive data are presented as means and standard deviations (SDs) (and medians and interquartile ranges [IQRs], as appropriate) for continuous variables and as percentages of participants for categorical variables. To minimize the influence of dietary misreporting [30] and to consider the differences in dietary intake owing to varying body sizes and energy requirements [31], free sugars intake was expressed as nutrient density. In addition to the total energy-adjusted intake of free sugars (% of energy intake, % E), the mean contribution of each food group (%) to free sugars intake was calculated. Based on the distribution of free sugars intake, participants were divided into quartiles for total free sugars [FS_total_] and FS_solids_. For FS_liquids_, participants were divided into tertiles for consumers and a separate group for non-consumers. In both sexes, approximately 60.0% of participants did not consume FS_liquids_, while all participants consumed FS_solids_ and were included in the FS_total_ category. The differences in basic characteristics according to the free sugars intake categories were determined using a linear trend test for continuous variables. Using general linear models, the adjusted mean intakes and standard errors for the BMI z-scores were estimated for each free sugars intake category. The Dunnett’s test was used to examine the differences among the free sugars intake categories, with the first (lowest) category as a reference. Linear regression analyses were also performed using the median value of each category of free sugars intake as a continuous variable. For the prevalence of overweight and obesity, odds ratios (ORs) and 95% confidence intervals (95% CIs) were estimated using logistic regression models with the first (lowest) category as a reference. The linear trend was tested using a logistic regression model with the median value of each free sugars intake category as a continuous variable. In the main analysis, three models were evaluated to consider the influence of energy intake and other macronutrients on weight status. In Model 1, adjustments were made for age (continuous). Model 2 was further adjusted for fat intake (% E, continuous) and dietary fibre intake (g/4184 kJ, continuous) [31]. Model 3 was further adjusted for energy intake (kJ, continuous) to investigate whether free sugars intake affected weight status through overconsumption of energy. Despite possible associations between physical activity and weight status and between socioeconomic status (SES) and free sugars intake [18], these factors were not treated as confounding factors and were not adjusted for. This was owing to the ordinal NHNS not having collected information on physical activity (e.g., habitual exercise and daily step count) and SES (e.g., household income and parental educational level and occupation) among child and adolescent participants [21,22].

### 2.5. Sensitivity Analysis

A sensitivity analysis was conducted, including participants whose height and weight were measured by trained fieldworkers (*n* = 1512, 54.4%) based on the same model as the main analysis. We further conducted an analysis by excluding participants who misreported their energy intakes (*n* = 65, 2.3%), as previously described [23]. Participants who were under-reporting their energy intakes selectively underreported their intake of confectioneries and soft drinks [32,33]. Additionally, participants with the lowest free sugar intakes more frequently underreported their energy intakes [34,35,36]. The misreporting of energy intake was assessed using the Goldberg cut-off method for the one-day dietary record data and the physical activity level for a sedentary lifestyle [37]. A plausible energy intake was defined as a reported energy intake to basal metabolic rate (BMR) ratio of 0.87–2.75. Based on age and body height, and weight, BMR was estimated using the sex- and age-specific equations by Henry [38] (for participants aged <18 years) or Ganpule [39,40] (for participants aged 18−19 years).

## 3. Results

The mean (SD) energy-adjusted intakes of FS_total_, FS_solids_, and FSl_iquids_ were 5.8 (4.4), 4.1 (2.9), and 1.8 (3.3) % E for males and 6.2 (4.4), 4.6 (3.1), and 1.6 (3.2) % E for females, respectively (Table 1). The medians (IQR) of corresponding intakes were 4.7 (2.8–7.8), 3.5 (2.0–5.4), and 0.0 (0.0–2.4) % E for males and 5.2 (3.1–8.3), 3.9 (2.3–6.1), and 0.0 (0.0–2.3) % E for females, respectively. The main contributors to FS_total_ were confectioneries, sugars and jams, seasonings, and soft drinks (83.9% for males and 84.4% for females). Consequently, the first three food groups were the major food sources of FS_solids_ (84.8% for males and 85.4% for females), whereas soft drinks were the top contributor to FS_liquids_ in both males (80.5%) and females (79.1%).

The mean ages (SD) were 10.4 (4.9) years for males and 9.9 (4.8) years for females, and the mean BMI scores (SD) were 17.8 (3.3) kg/m^2^ and 17.5 (2.8) kg/m^2^, respectively. Table 2 shows the basic characteristics of the participants according to their free sugars intake. For both males and females, participants with higher FS_total_ intakes had higher FS_solids_ and FS_liquids_ intakes. Similarly, those with higher FS_solids_ or FS_liquids_ intakes had higher FS_total_ intakes in both males and females. There was no association between FS_solids_ intake and FS_liquids_ intake, except that males with higher FS_liquids_ intakes were more likely to have higher FS_solids_ intakes. For both sexes, irrespective of the food sources, participants with higher free sugars intakes were more likely to be younger. However, no such association was noted with FS_liquids_ intakes in males. For energy intake, there was an inverse association with FS_solids_ and a positive association with FS_liquids_ in males.

Associations between free sugars intake and weight status are shown in Table 3 (using BMI z-scores) and Table 4 (using the prevalence of overweight and obesity). In males, the estimated BMI z-scores did not differ according to the free sugars intake categories for all food sources after adjustment for age and dietary intake (model 3, all *p* for trend ≥ 0.21). There were no substantial changes in the results when energy intake was not adjusted for (model 2, all *p* for trend ≥ 0.34). Similarly, in females, the estimated BMI z-scores did not differ according to the free sugars intake categories for FS_total_ and FS_liquids_, irrespective of whether energy intake was adjusted for (models 2 and 3, all *p* for trend ≥ 0.23). In contrast, females with higher FS_solids_ intakes tended to have lower estimated BMI z-scores after adjusting for energy intake (model 3, *p* for trend = 0.05). For both males and females, the estimated ORs and 95% CIs for the prevalence of overweight and obesity did not differ according to the free sugars intake categories for all food sources, irrespective of whether energy intake was adjusted for (models 2 and 3, all *p* trend ≥ 0.51).

The results of the sensitivity analysis for participants with measured anthropometry data were shown in Supplemental Tables S1 and S2. The results were generally the same as the main analyses. In males, no associations between free sugars intake and weight status were observed, irrespective of the source of free sugars and the indicator of weight status (i.e., z-scores or prevalence). In females, after adjustment for energy intake (model 3), higher FS_total_ intake was marginally associated with lower BMI z-scores (*p* for trend = 0.05), additionally to FS_solids_ intake (*p* for trend = 0.06) (Supplemental Table S1). Further, the ORs (95% CIs) for overweight and obese in the second and third categories of FS_solids_ intake were significantly lower than that in the first category after adjustment for energy intake (model 3: 0.45 [0.21, 0.99] and 0.39 [0.17, 0.88], *p* for trend = 0.26) (Supplemental Table S2). Meanwhile, the results of the analysis for participants with plausible energy intake were not substantially different from those of the primary analysis (data not shown).

## 4. Discussion

Here, for both males and females, free sugars intake from all food sources was not associated with BMI z-scores or the prevalence of overweight and obesity, except for a marginal association between higher FS_solids_ intakes and lower estimated BMI z-scores in females. To our knowledge, this was the first study to investigate the association between the food sources of free sugars and weight status among children and adolescents in Japan, using national survey data.

The mean FS_total_ intake in this study (5.8% E for males and 6.2% E for females) was compatible with those of previous studies conducted among children and adolescents in Japan (5.8–8.8% E) [19,20]. However, the present mean FS_total_ intake was lower than in Western countries, such as Australia and the United Kingdom (UK) (11.5−14.7% E [41] and 11.8−15.4% E [34]), and the United States (US) and Canada (14.8% E [12] and 12% E [13] as added sugars). Despite the relatively low mean intake, the major contributors to FS_total_ (confectioneries, sugars and jams, and soft drinks) among children and adolescents in Japan, in both the present and previous studies [20], were consistent with those of their Western counterparts [13,34,41]. However, the degree of contribution by soft drinks was lower among children and adolescents in Japan (15.5% for males and 13.2% for females in this study and 14.7–18.4% E in a previous study [20]) than by their counterparts in Western countries (free sugars, 17–33% in the UK [34] and added sugars, 18.0–33.9% in Australia [41], 12.3–53.3% in the US [42] and 22% in Canada [13]). Here, the lower contribution of soft drinks resulted in lower FS_liquids_ intakes (1.8% E for males and 1.6% E for females) than in previous studies conducted in the Western countries (added sugars from beverages, 6.9% E in the US [12]).

Compared with previous cross-sectional studies among children and adolescents in Japan [18,19], and in Australia [11], here, there was no association between FS_total_ intakes and weight status, irrespective of whether energy intake was adjusted for. A possible explanation is that the relatively low mean FS_total_ intake of the present population may be inadequate to influence the BMI z-scores and, consequently, the prevalence of overweight and obesity. Moreover, FS_liquids_ intakes were not associated with the BMI z-scores and the prevalence of overweight and obesity in this study, whether energy intake was adjusted for or not. These results are consistent with those of a previous cross-sectional study conducted in Australia [11] and a prospective design study conducted in Canada [14]. However, they are inconsistent with the results of cross-sectional studies conducted in America [12] and Canada [13], whereby added sugar intake from beverages was positively associated with weight status. Since sugars from beverages may be less satiating than those from solid food sources, they may cause an increase in energy intake and, therefore, weight gain [8,9]. Therefore, the lack of an association between FS_liquids_ intakes and BMI z-scores, and therefore the prevalence of overweight and obesity in this study, may be owing to the low mean FS_liquids_ intake. This may also explain the lack of an association between FS_total_ intakes and weight status, owing to the lower contribution of FS_liquids_ to FS_total_.

In contrast, after adjusting for energy intake, there was a marginal inverse association between FS_solids_ and the BMI z-scores in females. This association reached statistical significance when the analysis was restricted to participants with a plausible energy intake. A cross-sectional study that was conducted in America also reported an inverse association between weight status and added sugars from solid foods. However, the analysis was conducted among both sexes combined [12]. A cross-sectional design does not permit the assessment of causality owing to the uncertain temporality of the assessment. Therefore, these inverse associations may cause reverse causality; that is, participants with higher weight status may attempt to lose weight by reducing their consumption of sweet foods and beverages [43]. Here, the inconsistencies between males and females may be owing to gender differences in weight consciousness and dieting behaviors. Compared with males, females tend to perceive themselves as overweight and are more likely to be dieting, even at a young age [44]. Nonetheless, the present findings indicate that among children and adolescents in Japan, who have a relatively low mean intake of free sugars, consuming free sugars from any food source is unlikely to have an adverse effect on weight status. However, these results should not be misinterpreted as encouraging free sugars consumption, as these sugars will still contribute to energy intake as a macronutrient.

This study had several limitations. First, the one-day weighed household dietary records were insufficient to estimate the habitual dietary intake of individual participants owing to daily variation in dietary intake among free-living individuals. Additionally, differences in dietary intake between weekdays and weekend days and among seasons [45,46] could affect these results, as participants completed their dietary records on any weekday between October and November. When assessing the average dietary intake, these limitations may introduce bias and hamper the accuracy of these findings. However, in Japan, there is currently no nationally representative dietary survey based on multiple-day dietary assessment methods. When conducting future NHNS, the feasibility of a multiple-day dietary assessment covering all seasons and all the days of the week, or using a validated dietary assessment questionnaire, should be considered. Second, misreporting of energy intake is a common problem when performing dietary assessments, and this affects the accuracy of the relationship between energy intake and the variables of interest. However, the results of the sensitivity analysis excluding under- and over reporters (*n* = 65, 2.3%) indicated that the misreporting did not substantially influence the present findings nor change the conclusion of this analysis. Third, only half of the participants measured their body height and weight by trained fieldworkers (*n* = 1512, 54.4%). However, the results of the sensitivity analysis in such participants were almost similar to that of the total participants (i.e., marginal inverse associations between FS_total_ and FS_solids_ intakes and weight status in females). Therefore, any bias introduced by these survey methods was likely negligible for the present study. Fourth, as described in the methods section, physical activity was not adjusted for in this analysis. However, it is unlikely that participants with higher free sugars intakes had higher physical activity levels. Similarly, SES was not adjusted for despite its possible association with free sugars intake [18]. Hence, the present findings are not independent of the influence of these factors. When conducting future NHNS, collecting information on these factors among children and adolescent participants should be considered. Finally, while the NHNS aims to obtain a nationally representative sample of the non-institutionalized population of Japan, only 44.4% of the sampled households participated in the survey. Unfortunately, information on the characteristics of the participants in the households that refused to participate was not available, and the exact response rate of individual participants was not determined [21]. Additionally, the participants excluded from the analysis for missing information on body height or weight (*n* = 1 306) were more likely to be older. They had higher energy intakes (*p* ≤ 0.01) than those included in the analysis. Accordingly, a degree of selection bias could not be ruled out.

## 5. Conclusions

This national cross-sectional survey conducted among children and adolescents in Japan found no association between free sugars intake from all food sources and weight status, except for a marginal association between higher FS_solids_ intakes and lower estimated BMI z-scores in females. These findings indicate that among children and adolescents in Japan, who have a relatively low mean intake of free sugars, consuming free sugars from any food source is unlikely to have an adverse effect on weight status. However, this does not translate to encouraging excessive consumption of free sugars. Owing to the nature of a cross-sectional study design, it was impossible to establish a causal relationship in this analysis. Future prospective studies are necessary to confirm the relationship between the food sources of free sugars and the weight status identified in this study.

## Figures and Tables

**Table 1:** Contribution (%) of each food group to free sugars intake in Japanese children and adolescents aged 2–19 years: the 2016 National Health and Nutrition Survey, Japan.

	Males (*n* = 1438)						Females (*n* = 1340)					
	FS_total_		FS_solids_		FS_liquids_		FS_total_		FS_solids_		FS_liquids_	
	Mean	SD	Mean	SD	Mean	SD	Mean	SD	Mean	SD	Mean	SD
Intake (% E)	5.8	4.4	4.1	2.9	1.8	3.3	6.2	4.4	4.6	3.1	1.6	3.2
Contribution (%) ^1,2^												
Bread	0.1	1.4	0.1	1.5	-	-	0.2	2.0	0.2	2.1	-	-
Noodles	0.3	3.1	0.3	3.1	-	-	0.2	1.3	0.2	1.5	-	-
Other grain products	0.7	4.2	1.0	5.6	-	-	0.8	4.0	1.0	4.9	-	-
Potatoes	0.0	0.1	0.0	0.1	-	-	0.0	0.0	0.0	0.0	-	-
Sugars and jams	24.2	23.7	30.0	25.9	-	-	25.3	24.9	29.5	26.0	-	-
Pulses and nuts	0.5	3.8	0.6	4.0	-	-	0.3	2.5	0.4	2.8	-	-
Vegetables ^3^	0.7	3.2	0.8	3.4	-	-	0.5	2.6	0.6	2.7	-	-
Fruits	0.7	4.6	0.9	5.3	-	-	0.8	4.0	0.9	4.7	-	-
Fish and shellfish	2.8	7.6	3.6	9.0	-	-	2.8	7.4	3.5	8.4	-	-
Meats	2.0	4.8	2.4	5.7	-	-	1.9	5.3	2.3	5.7	-	-
Eggs	0.2	1.8	0.2	2.1	-	-	0.1	1.4	0.2	1.7	-	-
Dairy products	4.2	11.5	5.1	13.6	-	-	4.2	11.4	5.0	13.2	-	-
Fat and oil	0.2	1.0	0.3	1.2	-	-	0.3	1.2	0.4	1.6	-	-
Confectioneries	25.2	27.7	30.9	31.2	-	-	27.2	28.4	32.8	31.7	-	-
Fruit and vegetable juices	3.8	13.9	-	-	18.9	37.5	3.6	13.1	-	-	20.7	38.8
Alcoholic beverages	0.0	0.1	-	-	0.5	7.2	0.0	0.1	-	-	0.2	4.5
Soft drinks ^4^	15.5	25.5	-	-	80.5	38.0	13.2	23.6	-	-	79.1	39.0
Seasonings	19.0	20.8	23.8	22.4	-	-	18.7	20.4	23.2	22.4	-	-

FS_total_, total free sugars; FS_solids_, free sugars from solid foods; FS_liquids_, free sugars from beverages; SD, standard deviation; % E, percent of energy. ^1^ Estimated based on the intake of consumers: 1438 males and 1340 females for FS_total_ and FS_solids_; 584 males and 493 females for FS_liquids_. ^2^ Rice and grains and unsweetened tea and coffee were excluded due to the no contribution to free sugars intake. ^3^ Including mushrooms and seaweeds. ^4^ Including soda, sports drinks, fruit drinks, milk beverages, and pre-sweetened coffee.

**Table 2:** Basic characteristics of Japanese children and adolescents aged 2–19 years according to free sugars intake: the 2016 National Health and Nutrition Survey, Japan.

	Males (*n* = 1438)					Females (*n* = 1340)				
	First ^1^		Fourth ^1^		*p* ^2^	First ^1^		Fourth ^1^		*p* ^2^
**FS_total_**										
*n* (%)	359	(25.0)	359	(25.0)		335	(25.0)	335	(25.0)	
FS_total_ (% E)	1.6	0.7	11.8	4.1	<0.0001	1.8	0.7	12.2	4.2	<0.0001
FS_solids_ (% E)	1.6	0.7	6.6	3.8	<0.0001	1.7	0.8	7.7	3.6	<0.0001
FS_liquids_ (% E)	0.04	0.2	5.3	4.7	<0.0001	0.02	0.1	4.5	5.0	<0.0001
Age (years)	11.7	4.8	9.7	5.0	<0.0001	10.8	4.8	8.9	5.1	<0.0001
Energy intake (kJ)	8694	3088	8379	3052	0.23	6904	1790	6965	1970	0.76
Fat (% E)	30.1	6.6	28.0	6.0	<0.0001	31.0	6.3	28.7	6.2	<0.0001
Dietary fibre (g/4184 kJ)	6.1	1.9	5.7	1.8	<0.0001	6.5	1.9	6.3	1.9	0.09
**FS_solids_**										
*n* (%)	359	(25.0)	359	(25.0)		335	(25.0)	335	(25.0)	
FS_total_ (% E)	3.1	3.9	9.6	4.2	<0.0001	2.9	3.2	10.5	4.0	<0.0001
FS_solids_ (% E)	1.3	0.5	7.9	2.8	<0.0001	1.4	0.6	8.8	2.5	<0.0001
FS_liquids_ (% E)	1.8	3.8	1.8	3.0	0.97	1.52	3.2	1.7	3.1	0.68
Age (years)	11.6	4.9	9.1	4.6	<0.0001	10.9	4.6	8.9	4.9	<0.0001
Energy intake (kJ)	8565	3045	8140	2874	0.008	7055	1856	7005	1981	0.66
Fat (% E)	29.6	6.8	28.9	6.1	0.13	30.8	6.3	29.6	6.2	0.08
Dietary fibre (g/4184 kJ)	5.9	1.9	6.1	1.9	0.54	6.4	1.9	6.5	1.9	0.95
**FS_liquids_**										
*n* (%)	854	(59.4)	195	(13.6)		847	(63.2)	164	(12.2)	
FS_total_ (% E)	3.9	2.7	12.8	4.8	<0.0001	4.4	3.0	13.0	5.4	<0.0001
FS_solids_ (% E)	3.9	2.7	4.3	3.1	0.05	4.4	3.0	4.6	3.1	0.38
FS_liquids_ (% E)	0	0	8.5	4.0	<0.0001	0	0	8.3	4.6	<0.0001
Age (years)	10.5	4.9	10.8	5.3	0.59	10.4	4.9	9.1	5.1	<0.0001
Energy intake (kJ)	8280	2916	8889	3333	0.009	7043	1896	6947	2051	0.72
Fat (% E)	29.9	6.3	27.6	6.1	<0.0001	30.7	6.3	27.7	6.2	<0.0001
Dietary fibre (g/4184 kJ)	6.3	1.9	5.4	1.7	<0.0001	6.7	1.9	6.1	1.8	0.002

All values are means and standard deviations unless otherwise indicated. FS_total_, total free sugars; FS_solids_, free sugars from solid foods; FS_liquids_, free sugars from beverages; % E, percent of energy. ^1^ For FS_total_ and FS_solids_, the first to fourth categories consist of quartiles of participants. For FS_liquids_, the first category includes non-consumers, while the second to fourth categories consist of tertiles of consumers. ^2^ For continuous variables, a linear regression was used with the median value of each category of free sugars intake (FS_total_: 1.6, 3.8, 6.0, and 10.7% E for males and 1.7, 4.1, 6.5, and 10.8% E for females; FS_solids_: 1.4, 2.8, 4.3, and 7.1% E for males and 1.4, 3.1, 5.0, and 8.2% E for females and; FS_liquids_: 0, 1.4, 3.1, and 7.2% E for males and 0, 1.7, 3.1, and 6.6% E for females) as a continuous variable.

**Table 3:** BMI z-scores of Japanese children and adolescents aged 2–19 years according to free sugars intake: the 2016 National Health and Nutrition Survey, Japan ^1^.

	Males (*n* = 1438)									Females (*n* = 1340)								
	First ^2,3^		Second ^2,3^		Third ^2,3^		Fourth ^2,3^		*p* ^4^	First ^2,3^		Second ^2,3^		Third ^2,3^		Fourth ^2,3^		*p* ^4^
FS_total_																		
*n* (%)	359	(25.0)	360	(25.0)	360	(25.0)	359	(25.0)		335	(25.0)	335	(25.0)	335	(25.0)	335	(25.0)	
Intake (median, % E)	1.6		3.8		6.0		10.7			1.7		4.0		6.5		10.8		
Model 1 ^5^	0.05	0.06	−0.03	0.06	−0.05	0.06	−0.01	0.06	0.60	−0.04	0.05	−0.01	0.05	−0.07	0.05	−0.07	0.05	0.46
Model 2 ^6^	0.05	0.06	−0.02	0.06	−0.05	0.06	−0.01	0.06	0.46	−0.04	0.05	−0.01	0.05	−0.07	0.05	−0.07	0.05	0.46
Model 3 ^7^	0.07	0.06	−0.02	0.06	−0.06	0.06	−0.02	0.06	0.35	−0.01	0.05	−0.02	0.05	−0.08	0.05	−0.08	0.05	0.23
FS_solids_																		
*n* (%)	359	(25.0)	360	(25.0)	360	(25.0)	359	(25.0)		335	(25.0)	335	(25.0)	335	(25.0)	335	(25.0)	
Intake (median, % E)	1.4		2.8		4.3		7.1			1.4		3.1		5.0		8.2		
Model 1 ^5^	0.05	0.06	−0.04	0.06	0.01	0.06	−0.05	0.06	0.34	0.02	0.05	−0.09	0.05	0.00	0.05	−0.12	0.05	0.12
Model 2 ^6^	0.05	0.06	−0.04	0.06	0.01	0.06	−0.05	0.06	0.34	0.01	0.05	−0.09	0.05	0.00	0.05	−0.12	0.05	0.12
Model 3 ^7^	0.07	0.06	−0.04	0.06	0.00	0.06	−0.06	0.06	0.21	0.03	0.05	−0.09	0.05	−0.01	0.05	−0.13	0.05	0.05
FS_liquids_																		
*n* (%)	854	(59.4)	194	(13.5)	195	(13.6)	195	(13.6)		847	(63.2)	164	(12.2)	165	(12.3)	164	(12.2)	
Intake (median, % E)	0		1.4		3.1		7.2			0		1.7		3.1		6.6		
Model 1 ^5^	−0.02	0.04	0.07	0.08	−0.02	0.08	−0.02	0.08	0.95	−0.05	0.03	−0.03	0.07	−0.14	0.07	0.05	0.07	0.38
Model 2 ^6^	−0.02	0.04	0.08	0.08	−0.02	0.08	−0.02	0.08	0.95	−0.05	0.03	−0.04	0.07	−0.14	0.07	0.05	0.07	0.37
Model 3 ^7^	−0.01	0.04	0.04	0.08	−0.04	0.08	−0.03	0.08	0.69	−0.04	0.03	−0.08	0.07	−0.15	0.07	0.05	0.07	0.59

All values are adjusted means and standard errors unless otherwise indicated. FS_total_, total free sugars; FS_solids_, free sugars from solid foods; FS_liquids_, free sugars from beverages; BMI, body mass index; % E, percent of energy. ^1^ BMI z-scores were estimated based on BMI (calculated as kg/m^2^) using the International Obesity Task Force age- and sex-specific equations [28]. ^2^ For FS_total_ and FS_solids_, the first to fourth categories consist of quartiles of participants. For FS_liquids_, the first category includes non-consumers, while the second to fourth categories consist of tertiles of consumers. ^3^ Dunnett’s test was conducted using the first category as a reference. There was no difference between categories (*p* < 0.05). ^4^ A linear regression was conducted with the median value of each category of free sugars intake. ^5^ Adjustment was made for age (continuous). ^6^ Further adjustment was made for intakes of fat (% E, continuous) and dietary fiber (g/4184 kJ, continuous). ^7^ Further adjustment was made for energy intake (kJ, continuous).

**Table 4:** ORs (95% CIs) for overweight and obese Japanese children and adolescents aged 2–19 years according to free sugars intake: the 2016 National Health and Nutrition Survey, Japan ^1^.

	Males (*n* = 1438)								Females (*n* = 1340)							
	First ^2,3^	Second ^2,3^		Third ^2,3^		Fourth ^2,3^		*p* ^4^	First ^2,3^	Second ^2,3^		Third ^2,3^		Fourth ^2,3^		*p* ^4^
FS_total_																
Intake (median, % E)	1.6	3.8		6.0		10.7			1.7	4.0		6.5		10.8		
Overweight and obese (%)	12.8	10.0		7.8		12.8			8.1	6.0		5.4		8.4		
Model 1 ^5^	1.00	0.80	(0.50,1.27)	0.62	(0.38,1.03)	1.08	(0.70,1.69)	0.57	1.00	0.70	(0.38,1.27)	0.61	(0.33,1.13)	0.94	(0.54,1.64)	0.99
Model 2 ^6^	1.00	0.82	(0.52,1.32)	0.62	(0.38,1.03)	1.05	(0.67,1.66)	0.74	1.00	0.70	(0.39,1.29)	0.62	(0.33,1.15)	0.95	(0.54,1.68)	1.00
Model 3 ^7^	1.00	0.82	(0.51,1.31)	0.62	(0.37,1.02)	1.04	(0.66,1.65)	0.75	1.00	0.63	(0.34,1.16)	0.55	(0.30,1.03)	0.83	(0.47,1.49)	0.73
FS_solids_																
Intake (median, % E)	1.4	2.8		4.3		7.1			1.4	3.1		5.0		8.2		
Overweight and obese (%)	12.8	9.7		10.6		10.3			7.8	6.9		5.4		7.8		
Model 1 ^5^	1.00	0.75	(0.47,1.20)	0.87	(0.55,1.38)	0.87	(0.55,1.40)	0.77	1.00	0.85	(0.47,1.52)	0.62	(0.33,1.17)	0.89	(0.50,1.59)	0.69
Model 2 ^6^	1.00	0.80	(0.50,1.28)	0.89	(0.56,1.42)	0.89	(0.55,1.42)	0.77	1.00	0.88	(0.49,1.58)	0.63	(0.33,1.17)	0.91	(0.51,1.61)	0.69
Model 3 ^7^	1.00	0.79	(0.49,1.27)	0.88	(0.55,1.41)	0.88	(0.55,1.42)	0.76	1.00	0.83	(0.46,1.50)	0.58	(0.31,1.09)	0.83	(0.46,1.49)	0.51
FS_liquids_																
Intake (median, % E)	0.0	1.4		3.1		7.2			0.0	1.7		3.1		6.6		
Overweight and obese (%)	10.7	10.8		8.7		13.9			6.6	8.5		6.1		7.9		
Model 1 ^5^	1.00	1.03	(0.63,1.71)	0.83	(0.48,1.42)	1.32	(0.83,2.10)	0.34	1.00	1.26	(0.68,2.33)	0.84	(0.42,1.69)	1.14	(0.60,2.14)	0.81
Model 2 ^6^	1.00	1.04	(0.63,1.72)	0.80	(0.46,1.38)	1.24	(0.77,2.00)	0.51	1.00	1.25	(0.68,2.31)	0.85	(0.42,1.71)	1.14	(0.59,2.18)	0.80
Model 3 ^7^	1.00	1.03	(0.62,1.72)	0.80	(0.46,1.38)	1.24	(0.77,2.00)	0.52	1.00	1.08	(0.58,2.02)	0.79	(0.39,1.60)	1.06	(0.55,2.05)	0.99

ORs, odds ratios; CIs, confidence intervals % E, FS_total_, total free sugars; FS_solids_, free sugars from solid foods; FS_liquids_, free sugars from beverages; BMI, body mass index; % E, percent of energy. ^1^ Prevalence of overweight and obesity was estimated according to the International Obesity Task Force age- and sex-specific BMI (calculated as kg/m^2^) cut offs [28], which correspond to an adult BMI of ≥25 kg/m^2^, for subjects aged <18 years or based on BMI cut offs of ≥25 kg/m^2^ [29] for subjects aged 18–19 years. ^2^ For FS_total_ and FS_solids_, the first to fourth categories consist of quartiles of participants. For FS_liquids_, the first category includes non-consumers, while the second to fourth categories consist of tertiles of consumers. ^3^ ORs and 95% CIs were estimated by a logistic regression using the first category as a reference. ^4^ A logistic regression was conducted using the median value of each category of free sugars intake as a continuous variable ^5^ Adjustment was made for age (continuous). ^6^ Further adjustment was made for intakes of fat (% E, continuous) and dietary fibre (g/4184 kJ, continuous). ^7^ Further adjustment was made for energy intake (kJ, continuous).

## Data Availability

Data cannot be shared because the MHLW permits individuals who applied for secondary use of the NHNS data to refer to the data.

## Supplementary Materials

The following are available online at https://www.mdpi.com/article/10.3390/nu14173659/s1, Table S1: BMI z-scores of Japanese children and adolescents aged 2–19 years with measured anthropometric data according to free sugars intake: the 2016 National Health and Nutrition Survey, Japan. Table S2: ORs (95% CIs) for overweight and obese Japanese children and adolescents aged 2–19 years with measured anthropometric data according to free sugars intake: the 2016 National Health and Nutrition Survey, Japan.
