# Supplementary material for: Association between Food Sources of Free Sugars and Weight Status among Children and Adolescents in Japan: The 2016 National Health and Nutrition Survey, Japan
Source: Nutrients. 2022 Sep 4;14(17):3659. doi: 10.3390/nu14173659 (PMC9460394; doi:10.3390/nu14173659)
Supplement: Supplementary file 1 [file nutrients-14-03659-s001.zip › nutrients-1878285-supplementary.pdf]

Supplemental Table S1. BMI z-scores of Japanese children and adolescents aged 2-19 years with measured anthropometric data according to free sugars intake: the 2016 National Health and Nutrition Survey, Japan [1].

	Males (n = 776)									Females (n = 736)								
	First [2,3]		Second [2,3]		Third [2,3]		Fourth [2,3]		P [4]	First [2,3]		Second [2,3]		Third [2,3]		Fourth [2,3]		P [4]
FS_{total}																		
n (%)	194	(25.0)	194	(25.0)	194	(25.0)	194	(25.0)		184	(25.0)	184	(25.0)	184	(25.0)	184	(25.0)	
Intake (median, %E)	1.8		3.9		6.0		10.7			1.9		4.4		7.0		11.7		
Model 1 [5]	0.07	0.08	-0.04	0.08	-0.07	0.08	-0.01	0.08	0.64	0.06	0.07	0.05	0.07	-0.08	0.07	-0.09	-0.09	0.07
Model 2 [6]	0.05	0.08	-0.03	0.08	-0.07	0.08	0.00	0.08	0.86	0.06	0.07	0.05	0.07	-0.08	0.07	-0.09	-0.09	0.08
Model 3 [7]	0.07	0.08	-0.03	0.08	-0.09	0.08	0.01	0.08	0.83	0.07	0.07	0.05	0.07	-0.08	0.07	-0.09	-0.09	0.05
FS_{solids}																		
n (%)	194	(25.0)	194	(25.0)	194	(25.0)	194	(25.0)		184	(25.0)	184	(25.0)	184	(25.0)	184	(25.0)	
Intake (median, %E)	1.4		3.0		4.5		7.5			1.5		3.3		5.2		8.7		
Model 1 [5]	0.08	0.08	0.01	0.08	-0.07	0.08	-0.06	0.08	0.18	0.15	0.07	-0.08	0.07	-0.06	0.07	-0.07	-0.07	0.08
Model 2 [6]	0.07	0.08	0.00	0.08	-0.07	0.08	-0.06	0.08	0.24	0.14	0.07	-0.08	0.07	-0.06	0.07	-0.06	-0.06	0.08
Model 3 [7]	0.09	0.08	0.00	0.08	-0.08	0.08	-0.06	0.08	0.18	0.15	0.07	-0.08	0.07	-0.06	0.07	-0.07	-0.07	0.06
$FS_{liquids}$																		
n (%)	466	(60.1)	103	(13.3)	104	(13.4)	103	(13.3)		450	(61.1)	95	(12.9)	96	(13.0)	95	(12.9)	
Intake (median, %E)	0		1.2		2.9		6.6			0		1.7		3.2		6.7		
Model 1 [5]	-0.01	0.05	0.04	0.11	-0.09	0.11	0.01	0.11	0.99	0.03	0.04	-0.03	0.09	-0.14	0.09	-0.06	-0.06	0.22
Model 2 [6]	-0.01	0.05	0.03	0.11	-0.09	0.11	0.02	0.11	0.92	0.02	0.04	-0.03	0.09	-0.14	0.09	-0.05	-0.05	0.24
Model 3 [7]	-0.005	0.05	0.004	0.11	-0.11	0.11	0.04	0.11	0.90	0.03	0.04	-0.06	0.09	-0.14	0.09	-0.06	-0.06	0.19

All values are adjusted means and standard errors unless otherwise indicated.

FS_{total}, total free sugars; FS_{solids}, free sugars from solid foods; $FS_{liquids}$, free sugars from beverages; BMI, body mass index; %E, percent of energy

[1] BMI z-scores were estimated based on BMI (calculated as kg/m^2) using the International Obesity Task Force age- and sex-specific equations.

[2] For FS_{total} and FS_{solids}, the first to fourth categories consist of quartiles of participants. For $FS_{liquids}$, the first category includes non-consumers, while the second to fourth categories consist of tertiles of consumers.

[3] Dunnett's test was conducted using the first category as a reference. There was no difference between categories ($P < 0.05$).

[4] A linear regression was conducted with the median value of each category of free sugars intake.

[5] Adjustment was made for age (continuous).

[6] Further adjustment was made for intakes of fat (%E, continuous) and dietary fibre (g/4184 kJ, continuous).

[7] Further adjustment was made for energy intake (kJ, continuous).

Supplemental Table S2. ORs (95% CIs) for overweight and obese Japanese children and adolescents aged 2-19 years with measured anthropometric data according to free sugars intake: the 2016 National Health and Nutrition Survey, Japan [1].

	Males (n = 776)								Females (n = 736)							
	First [2,3]	Second [2,3]		Third [2,3]		Fourth [2,3]		P [4]	First [2,3]	Second [2,3]		Third [2,3]		Fourth [2,3]		P [4]
FS_{total}																
Intake (median, %E)	1.8	3.9		6.0		10.7			1.9	4.4		7.0		11.7		
Overweight and obese (%)	11.9	12.4		7.7		10.8			10.3	5.9		7.6		5.4		
Model 1 [5]	1.00	1.19	(0.64, 2.22)	0.70	(0.35, 1.40)	1.06	(0.56, 2.02)	0.91	1.00	0.54	(0.25, 1.18)	0.70	(0.34, 1.44)	0.48	(0.22, 1.07)	0.12
Model 2 [6]	1.00	1.29	(0.69, 2.42)	0.73	(0.36, 1.46)	1.18	(0.61, 2.28)	0.91	1.00	0.55	(0.25, 1.20)	0.69	(0.33, 1.43)	0.46	(0.20, 1.05)	0.10
Model 3 [7]	1.00	1.29	(0.69, 2.42)	0.73	(0.36, 1.46)	1.18	(0.61, 2.28)	0.90	1.00	0.53	(0.24, 1.15)	0.66	(0.32, 1.38)	0.44	(0.19, 1.01)	0.09
FS_{solids}																
Intake (median, %E)	1.4	3.0		4.5		7.5			1.5	3.3		5.2		8.7		
Overweight and obese (%)	10.8	14.4		9.3		8.3			11.4	5.4		4.9		7.6		
Model 1 [5]	1.00	1.49	(0.81, 2.74)	0.92	(0.47, 1.81)	0.86	(0.43, 1.72)	0.35	1.00	0.44	(0.20, 0.97)	0.39	(0.18, 0.89)	0.62	(0.30, 1.27)	0.29
Model 2 [6]	1.00	1.53	(0.83, 2.83)	0.97	(0.50, 1.91)	0.92	(0.46, 1.86)	0.48	1.00	0.46	(0.21, 1.01)	0.40	(0.18, 0.90)	0.62	(0.30, 1.29)	0.28
Model 3 [7]	1.00	1.55	(0.83, 2.87)	0.98	(0.50, 1.94)	0.93	(0.46, 1.88)	0.49	1.00	0.45	(0.21, 0.99)	0.39	(0.17, 0.88)	0.61	(0.29, 1.26)	0.26
$FS_{liquids}$																
Intake (median, %E)	0	1.2		2.9		6.6			0	1.7		3.2		6.7		
Overweight and obese (%)	10.9	10.7		5.8		14.6			8.4	7.4		5.2		4.2		
Model 1 [5]	1.00	1.00	(0.50, 2.00)	0.52	(0.22, 1.25)	1.47	(0.79, 2.75)	0.43	1.00	0.86	(0.37, 1.98)	0.58	(0.22, 1.53)	0.47	(0.16, 1.34)	0.10
Model 2 [6]	1.00	0.95	(0.47, 1.91)	0.52	(0.21, 1.26)	1.53	(0.80, 2.91)	0.37	1.00	0.83	(0.36, 1.92)	0.58	(0.22, 1.51)	0.43	(0.15, 1.25)	0.07
Model 3 [7]	1.00	0.95	(0.47, 1.92)	0.52	(0.21, 1.26)	1.53	(0.80, 2.92)	0.37	1.00	0.78	(0.33, 1.82)	0.57	(0.22, 1.49)	0.42	(0.14, 1.23)	0.07

ORs, odds ratios; CIs, confidence intervals %E, FS_{total}, total free sugars; FS_{solids}, free sugars from solid foods; $FS_{liquids}$, free sugars from beverages; BMI, body mass index; %E, percent of energy

[1] Prevalence of overweight and obesity was estimated according to the International Obesity Task Force age- and sex-specific BMI (calculated as kg/m^2) cut offs, which correspond to an adult BMI of ≥25 kg/m^2, for subjects aged <18 years or based on BMI cut offs of ≥25 kg/m^2 for subjects aged 18-19 years.

[2] For FS_{total} and FS_{solids}, the first to fourth categories consist of quartiles of participants. For $FS_{liquids}$, the first category includes non-consumers, while the second to fourth categories consist of tertiles of consumers.

[3] ORs and 95% CIs were estimated by a logistic regression using the first category as a reference.

[4] A logistic regression was conducted using the median value of each category of free sugars intake as a continuous variable

[5] Adjustment was made for age (continuous).

[6] Further adjustment was made for intakes of fat (%E, continuous) and dietary fibre (g/4184 kJ, continuous).

[7] Further adjustment was made for energy intake (kJ, continuous).
